# Supplementary material for: Role of Human Papillomavirus Infection in Head and Neck Cancer in Italy: The HPV-AHEAD Study
Source: Cancers (Basel). 2020 Nov 29;12(12):3567. doi: 10.3390/cancers12123567 (PMC7760748; doi:10.3390/cancers12123567)
Supplement: Supplementary file 1 [file cancers-12-03567-s001.pdf]

Supplementary Materials:

Role of Human Papillomavirus Infection in Head and Neck Cancer in Italy: The HPV-AHEAD Study

Marta Tagliabue, Marisa Mena, Fausto Maffini, Tarik Gheit, Beatriz Quirós Blasco, Dana Holzinger, Sara Tous, Daniele Scelsi, Debora Riva, Enrica Grosso, Francesco Chu, Eric Lucas, Ruediger Ridder, Susanne Rehm, Johannes Paul Bogers, Daniela Lepanto, Belén Lloveras Rubio, Rekha Vijay Kumar, Nitin Gangane, Omar Clavero, Michael Pawlita, Devasena Anantharaman, Madhavan Radhakrishna Pillai, Paul Brennan, Rengaswamy Sankaranarayanan, Marc Arbyn, Francesca Lombardi, Miren Taberna, Sara Gandini, Fausto Chiesa, Mohssen Ansarin, Laia Alemany, Massimo Tommasino, Susanna Chiocca and the HPV-AHEAD Study Group

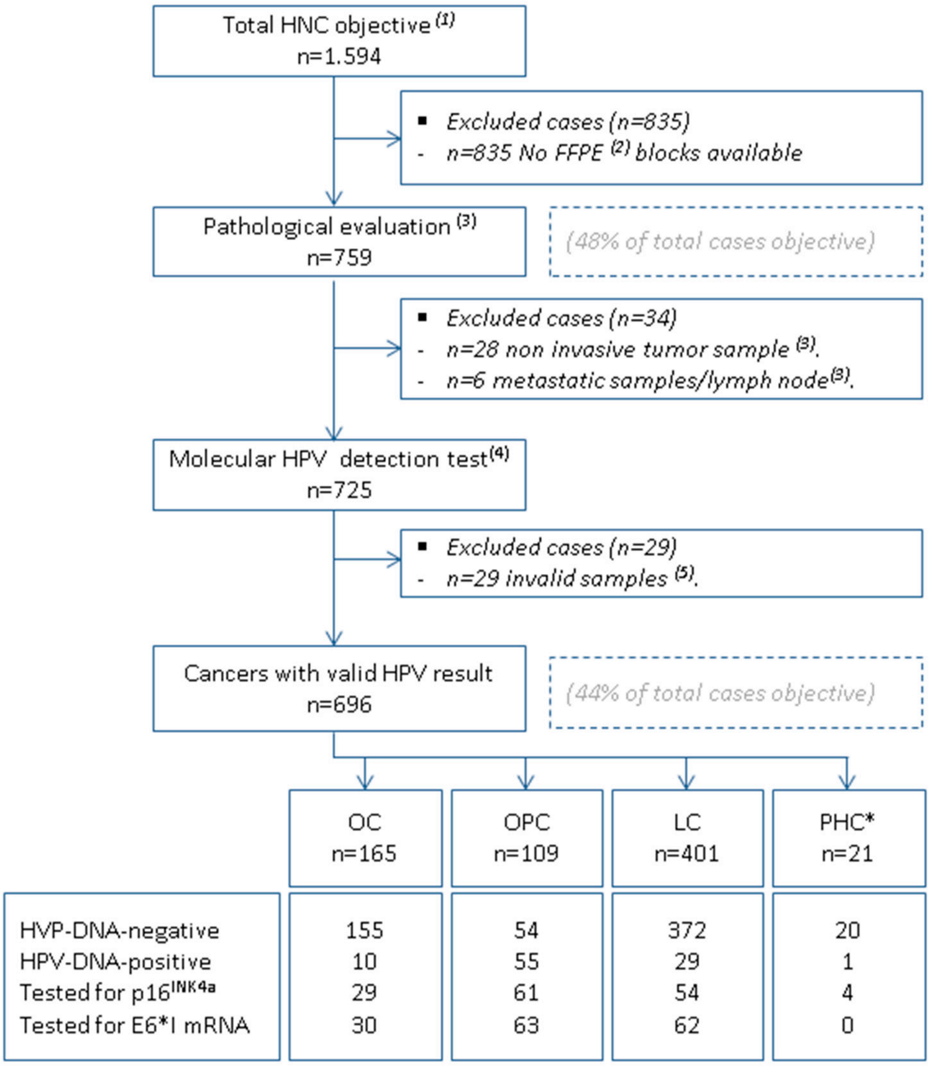

OC: Oral cavity cancer; OPC: Oropharyngeal cancer; LC: Laryngeal cancer: PHC: Pharyngeal-Hypopharyngeal cancer.
** PHC were finally excluded from the analyses.*

(1) *Total HNC (Head and Neck cancer cases) diagnosed between 2000 to 2010 in IEO, Milan, Italy.*
(2) *FFPE (formalin-fixed paraffin-embedded) blocks available: biopsy or surgical piece from HNC patients.*
(3) *Histopathological evaluation of HE (Hematoxylin and Eosin) sample under a form agreed by a panel of expert pathologists.*
(4) *HPV molecular test detection: DNA detection test, p16^INK4a expression, RNA detection test.*
(5) *Invalid samples in any of the quality controls of HPV molecular detection techniques: information provided in Molecular Data Base. Molecular techniques for HPV detection and their quality control:*
   - *HPV-DNA detection using Luminex Technology where two primers for amplification of the beta-globin gene were also included to control for the quality of the template DNA;*
   - *HPV E6*I mRNA with available material that tested positive for an HPV type for which the type-specific mRNA detection. Tested in an random HPV-DNA- cases (13% OC, 15%OPC, 9%LC, 15% PHC). Used Ubiquitine as a quality control.*
   - *p16^INK4a with their quality tissue control. Tested in HPV-DNA+ samples with available material and in a random of HPV-DNA- cases (12% OC, 15%OPC, 9%LC, 15% PHC).*

Figure S1. Flowchart of cases.

Table S1. Association of demographics and clinical characteristics of OC cancers and HPV positivity according to three different HPV-relatedness definitions: HPV-DNA, HPV-E6*I mRNA detection, and HPV-DNA/p16^{INK4a}.

**Characteristics**	**OC samples**	**HPV-DNA detection**			**HPV-E6*I mRNA detection**			**HPV-DNA AND p16^{INK4a} detection**		
	**(n = 165)**	**Positive**	**OR crude**	**OR adjusted[c]**	**Positive**	OR crude	OR adjusted[c]	Positive	OR crude	OR adjusted[c]
	No. (%)[a]	(n = 10) No. (%)[b]	(95%CI)	(95%CI)	(n = 3) No. (%)[b]	(95%CI)	(95%CI)	(n =4) No. (%)[b]	(95%CI)	(95%CI)
**Age at diagnosis**			0.99 (0.95–1.03)			1.02 (0.95–1.10)			0.98 (0.93–1.05)	
17–54 y	71 (43.0)	5 (7.0)	Ref.		1 (1.4)	Ref.		2 (2.8)	Ref.	
55–62 y	28 (17.0)	2 (7.1)	1.01 (0.9-5.57)		0 (0.0)	–		0 (0.0)	–	
63–70 y	27 (16.4)	2 (7.4)	1.06 (0.19-5.80)		2 (7.4)	5.60 (0.49–64.47)		2 (7.4)	2.76 (0.37–20.65)	
71–94 y	39 (23.6)	1 (2.5)	0.34 (0.04-3.08)		0 (0.0)	–		0 (0.0)	–	
**Gender**										
Male	99 (60.0)	6 (6.1)	Ref.		2 (2.0)	Ref.		3 (3.1)	Ref.	
Female	66 (40.0)	4 (6.1)	1.00 (0.27–3.69)		1 (1.5)	0.75 (0.07–8.39)		1 (1.5)	0.48 (0.05–4.79)	
**Period of diagnosis**			1.19 (0.95–1.50)			1.19 (0.79–1.78)			1.45 (0.82–1.62)	
2000–2003	49 (29.7)	1 (2.0)	Ref.		0 (0.0)	–		0 (0.0)	–	

2004–2007	68 (41.2)	5 (7.4)	3.81 (0.43–33.69)		2 (2.9)	1.42 (0.13–16.17)		3 (4.5)	2.20 (0.322–21.84)
2008–2010	48 (29.1)	4 (8.3)	4.36 (0.47–40.55)		1 (2.0)	Ref.		1 (2.1)	Ref.
**Tobacco behaviour**									
Non-smoker	62 (37.6)	4 (6.5)	Ref.		1 (1.6)	Ref.		1 (1.6)	Ref.
Former smoker	31 (18.8)	2 (6.5)	1.00 (0.17–5.78)		2 (6.5)	4.21 (0.37–48.31)		2 (6.5)	4.21 (0.37-48.31)
Smoker	63 (38.2)	2 (3.2)	0.48 (0.08–2.69)		0 (0.0)	–		0 (0.0)	–
Unknown	9 (5.4)	2 (22.2)			0 (0.0)			1 (11.1)	
**Alcohol behaviour**									
Non-drinker	67 (40.6)	5 (7.5)	2.18 (0.50–9.46)		1 (1.5)	0.62 (0.05–7.00)		1 (1.5)	Ref.
Former drinker	3 (1.8)	0 (0.0)	–		0 (0.0)	–		0 (0.0)	–
Drinker	84 (50.9)	3 (3.6)	Ref.		2 (2.4)	Ref.		2 (2.4)	1.63 (0.14–18.37)
Unknown	11 (6.7)	2 (18.2)			0 (0.0)			1 (9.1)	
**Subsite**									
Proximal to oropharynx	50 (30.3)	6 (12.0)	**3.78 (1.02–14.06)**	**3.78 (1.02–14.06)**	2 (4.0)	4.75 (0.42–53.63)		2 (4.0)	2.40 (0.33–17.58)
Distal to oropharynx	115 (69.7)	4 (3.5)	Ref.	Ref.	1 (0.9)	Ref.		2 (1.7)	Ref.

**Stage (7th edition TNM)**							
I	52 (31.5)	4 (7.7)	Ref.	1 (1.9)	Ref.	1 (1.9)	Ref.
II	22 (13.3)	1 (4.5)	0.57 (0.06–5.42)	0 (0.0)	–	0 (0.0)	–
III	28 (17.0)	2 (7.1)	0.92 (0.16–5.38)	0 (0.0)	–	1 (3.7)	1.96 (0.11–32.64)
IVa	62 (37.6)	3 (4.8)	0.61 (0.13–2.86)	2 (3.2)	1.70 (0.15–19.30)	2 (3.2)	1.70 (0.15–19.29)
IVb	0 (0.0)	–		–		–	
IVc	1 (0.6)	0 (0.0)	–	0 (0.0)	–	0 (0.0)	–
**Treatment**							
Only surgery	85 (51.5)	5 (5.9)	1.42 (0.33–6.15)	1 (1.2)	0.83 (0.05–13.571)	1 (1.2)	Ref.
Surgery + others	71 (43.0)	3 (4.2)	Ref.	1 (1.4)	Ref.	2 (2.8)	2.41 (0.22–27.42)
No surgery	2 (1.2)	2 (100.0)	–	1 (50.0)	70.00 (2.33–2103.43)	1 (100.0)	–
Only radio	1 (0.6)	0 (0.0)	–	0 (0.0)		0 (0.0)	–
Unknown	6 (3.6)	0 (0.0)		0 (0.0)		0 (0.0)	
**Histological diagnosis**							
SCC Conventional keratinizing	112 (67.9)	5 (4.5)	Ref.	0 (0.0)	–	1 (0.9)	Ref.

SCC Conventional non ker.	47 (28.5)	5 (10.6)	2.55 (0.70–9.25)	3 (6.4)		3 (6.5)	7.74 (0.78–76.50)
SCC Other	6 (3.6)	0 (0.0)	–	0 (0.0)	–	0 (0.0)	–
Other non-SCC	0 (0.0)	–					
**Tumour differentiation**							
Grade 1	72 (43.6)	5 (6.9)	Ref.	0 (0.0)		1 (1.4)	Ref.
Grade 2	54 (32.7)	1 (1.9)	0.25 (0.03–2.23)	1 (1.9)	Ref.	1 (1.9)	1.33 (0.08–21.91)
Grade 3	38 (20.3)	4 (10.5)	1.60 (0.40–6.25)	2 (5.3)	2.94 (0.26–33.7)	2 (5.4)	4.06 (0.35–46.28)
Unknown	1 (0.6)	0 (0.0)		0 (0.0)		0 (0.0)	
**Total**	165 (100.0)	10 (6.1)		3 (1.8)		4 (2.4)	

OC: oral cavity cancer; OR: odds ratio; Distal to oropharynx: C02 and C02.0 and C02.1 and C02.2 and C02.3 and C03.1 and C04.1 and C04.9 and C06.0; Proximal to oropharynx: C02.8 and C02.9 and C05.8 and C06.2; [a] column percentage. [b] row Percentage. [c] adjusted logistic regressions model using Backward Method initially including variables with crude p-value ≤ 0.25, and finally selecting variables with p-value < 0.05; bold represents statistically significant categories.

Table S2. Association of demographics and clinical characteristics of OPC cancers and HPV positivity according to three different HPV-relatedness definitions: HPV-DNA, HPV-E6*I mRNA detection, and HPV-DNA/p16$^{INK4a.}$

Characteristics	OPC samples (n = 109) No. (%)[a]	HPV-DNA detection Positive (n = 55) No. (%)[b]	OR crude (95%CI)	OR adjusted[c] (95%CI)	HPV-E6*I mRNA detection Positive (n = 44) No. (%)[b]	OR crude (95%CI)	OR adjusted[c] (95%CI)	HPV-DNA AND p16^{INK4a} detection Positive (n =47) No. (%)[b]	OR crude (95%CI)	OR adjusted[c] (95%CI)
**Age at diagnosis**			0.97 (0.93–1.01)	**0.94 (0.90–0.99)**		1.00 (0.96–1.04)			0.99 (0.95–1.02)	
17–54 y	25 (22.9)	17 (68.0)	**Ref.**		11 (44.0)	**Ref.**		14 (56.0)	**Ref.**	
55–62 y	38 (34.8)	19 (50.0)	0.47 (0.16-1.34)		15 (39.5)	0.83 (0.29–2.31)		16 (43.2)	0.59 (0.22–1.66)	
63–70 y	24 (22.0)	9 (37.5)	0.28 (0.07-0.92)		8 (33.3)	0.63 (0.19–2.03)		8 (33.3)	0.39 (0.12–1.25)	
71–94 y	22 (20.2)	10 (45.5)	0.39 (0.12-1.28)		10 (45.5)	1.06 (0.34–3.36)		9 (42.9)	0.59 (0.18–1.89)	
**Gender**										
Male	85 (78.0)	42 (49.4)	**Ref.**		34 (40.0)	**Ref.**		37 (44.1)	**Ref.**	

Female	24 (22.0)	13 (54.2)	1.21 (0.49–3.00)		10 (41.7)	1.07 (0.43–2.69)		10 (43.5)	0.97 (0.38–2.48)	
**Period of diagnosis**			**1.19 (1.04–1.35)**	**1.21 (1.05–1.39)**		**1.22 (1.07–1.40)**	**1.24 (1.07–1.44)**		**1.21 (1.06–1.38)**	**1.22 (1.06–1.42)**
2000–2003	41 (37.3)	14 (34.2)	**Ref.**		10 (24.4)	**Ref.**		10 (25.0)	**Ref.**	
2004–2007	46 (42.7)	26 (56.5)	**2.51 (1.05–5.98)**		20 (43.5)	2.38 (0.95–5.99)		24 (52.2)	**3.27 (1.30–8.21)**	
2008–2010	22 (20.0)	15 (68.2)	**4.13 (1.37–12.48)**		14 (63.6)	**5.43 (1.76–16.69)**		13 (61.9)	**4.85 (1.57–15.16)**	
**Tobacco behaviour**										
Non-smoker	21 (19.3)	13 (61.9)	**3.35 (1.17–9.60)**	**3.95 (1.26–12.39)**	12 (57.1)	**4.97 (1.67–14.79)**	**5.15 (1.65–16.08)**	13 (61.9)	**5.76 (1.91–17.41)**	**5.91 (1.88–18.65)**
Former smoker	22 (20.2)	16 (72.3)	**5.49 (1.82–16.54)**	**7.22 (2.15–24.21)**	13 (59.1)	**5.38 (1.83–15.85)**	**5.32 (1.65–10.08)**	15 (68.2)	**7.59 (2.48–23.26)**	**7.69 (2.36–25.05)**
Smoker	52 (47.7)	17 (32.7)	**Ref.**	**Ref.**	11 (21.2)	**Ref.**	**Ref.**	11 (22.0)	**Ref.**	**Ref.**
Unknown	14 (12.8)	9 (64.3)			8 (57.1)			8 (57.1)		
**Alcohol behaviour**										

Non-drinker	26 (23.8)	15 (57.7)	1.59 (0.64–4.02)	12 (46.2)	1.49 (0.59–3.76)	14 (53.8)	1.92 (0.76–4.88)
Former drinker	3 (2.8)	0 (0.0)	–	0 (0.0)	–	0 (0.0)	–
Drinker	63 (57.8)	29 (46.0)	**Ref.**	23 (36.5)	**Ref.**	23 (37.7)	**Ref.**
Unknown	17 (15.6)	11 (64.7)		9 (52.9)		10 (58.8)	
**Subsite**							
Tonsil	49 (45.0)	30 (61.2)	**3.32 (1.29–8.55)**	27 (55.1)	**5.11 (1.78–14.67)**	27 (56.3)	**5.14 (1.78–14.86)**
BOT	28 (26.6)	15 (51.7)	2.25 (0.79–6.41)	11 (37.9)	2.54 (0.79–8.16)	14 (48.3)	**3.73 (1.18–11.83)**
Other oropharynx	31 (28.4)	10 (32.3)	**Ref.**	6 (19.4)	**Ref.**	6 (20.0)	**Ref.**
**Stage (7th edition TNM)**							
I	12 (11.0)	7 (58.3)	**Ref.**	3 (25.0)	**Ref.**	4 (33.3)	**Ref.**
II	12 (11.0)	4 (33.3)	0.36 (0.07–1.88)	3 (25.0)	1.00 (0.15–6.34)	3 (25.0)	0.67 (0.11–3.93)
III	22 (20.2)	13 (59.1)	1.03 (0.25–4.30)	12 (54.6)	3.60 (0.76–17.01)	11 (55.0)	2.44 (0.55–10.83)

IVa	51 (46.8)	27 (52.9)	0.80 (0.23–2.87)	22 (43.1)	2.28 (0.55–9.41)	25 (49.0)	1.92 (0.51–7.20)
IVb	8 (7.3)	3 (37.5)	0.43 (0.07–2.68)	3 (37.5)	1.80 (0.26–12.5)	3 (37.5)	1.20 (0.19–7.77)
IVc	4 (3.7)	1 (25.0)	0.24 (0.02–3.01)	1 (25.0)	1.00 (0.07–13.64)	1 (25.0)	0.67 (0.05–8.64)
**Treatment**							
Only surgery	22 (20.2)	8 (36.4)	1.33 (0.27–6.65)	3 (13.6)	0.37 (0.06–2.27)	6 (27.3)	0.88 (0.17–4.54)
Surgery + others	41 (37.6)	20 (48.8)	2.22 (0.50–9.81)	16 (39.0)	1.49 (0.34–6.63)	18 (43.9)	1.83 (0.41–8.07)
No surgery	29 (26.6)	20 (69.0)	**5.19 (1.08–24.79)**	19 (65.5)	4.43 (0.94–20.98)	17 (62.9)	3.97 (0.83–18.91)
Only radio	10 (9.2)	3 (30.0)	**Ref.**	3 (30.0)	Ref.	3 (30.0)	**Ref.**
Unknown	7 (6.4)	4 (57.1)		3 (42.9)		3 (42.9)	
**Histological diagnosis**							
SCC Conventional keratinizing	28 (25.7)	10 (35.7)	**Ref.**	2 (7.1)	**Ref.**	4 (14.3)	**Ref.**

SCC Conventional non ker.	52 (47.7)	23 (44.2)	1.43 (0.55–3.68)	22 (42.3)	**9.53 (2.04–44.46)**	22 (43.1)	**4.55 (1.38–15.03)**
SCC Other	22 (20.2)	18 (81.8)	**8.10 (2.14–30.65)**	16 (72.7)	**34.67 (6.22–193.06)**	17 (80.9)	25.50 (5.58–116.45)
Other non-SCC	7 (6.4)	4 (57.1)	2.40 (0.45–12.94)	4 (57.1)	**17.33 (2.17–138.18)**	4 (57.1)	8.00 (1.28–50.04)
**Tumour differentiation**							
Grade 1	9 (8.3)	3 (33.3)	Ref.	0 (0.0)	–	1 (11.1)	Ref.
Grade 2	30 (27.5)	16 (53.3)	2.29 (0.48–10.88)	11 (36.7)	Ref.	13 (43.3)	6.12 (0.68–55.25)
Grade 3	67 (61.5)	34 (50.8)	2.06 (0.48–8.93)	31 (46.3)	1.49 (0.61–3.60)	32 (48.4)	7.53 (0.89–63.62)
Unknown	3 (2.7)	2 (66.7)		2 (66.7)		1 (50.0)	
**Total**	109 (100.0)	55 (50.5)		44 (40.4)		47 (43.9)	

OPC: oropharyngeal cancer; OR: odds ratio; Tonsil: C02.4 and C09.0 and C09.1 and C09.9; BOT: base of the tongue (C01); Other oropharynx: C10 and C10.0 and C10.2 and C10.3 and C10.8 and C10.9; [a] column percentage; [b] row percentage; [c] adjusted logistic regressions model using Backward Method initially including variables with crude p-value ≤ 0.25, and finally selecting variables with p-value < 0.05; bold represents statistically significant categories.

Table S3. Association of demographics and clinical characteristic of LC cancers and HPV positivity according to three different HPV-relatedness definitions: HPV-DNA, HPV-E6*I mRNA detection, and HPV-DNA/p16^{INK4a}.

	LC samples	HPV-DNA detection			HPV- E6*I mRNA detection			HPV-DNA AND p16^{INK4a} detection		
**Characteristics**	**(n = 401)**	**Positive**	**OR crude**	**OR adjusted**[c]	**Positive**	**OR crude**	**OR adjusted**[c]	**Positive**	**OR crude**	**OR adjusted**[c]
	No. (%)[a]	(n = 29) No. (%)[b]	(95%CI)	(95%CI)	(n = 9) No. (%)[b]	(95%CI)	(95%CI)	(n =7) No. (%)[b]	(95%CI)	(95%CI)
**Age at diagnosis**			0.98 (0.94–1.01)			**0.88 (0.82–0.95)**	**0.87 (0.80–0.94)**		**0.90 (0.83–0.97)**	
17–54 y	70 (17.5)	9 (12.9)	1.84 (0.67–5.03)		5 (7.1)	8.23 (0.94–72.02)		4 (5.8)	6.58 (0.72–60.19)	
55–62 y	115 (28.7)	9 (7.8)	1.06 (0.39–2.86)		3 (2.6)	2.87 (0.29–27.98)		2 (1.7)	1.89 (0.17–21.19)	
63–70 y	108 (26.9)	3 (2.8)	0.36 (0.92–1.38)		0 (0.0)	**–**		0 (0.0)	**–**	
71–94 y	108 (26.9)	8 (7.4)	Ref.		1 (0.9)	**Ref.**		1 (0.9)	**Ref.**	
**Gender**										
Male	355 (88.5)	25 (7.0)	Ref.		6 (1.7)	Ref.		5 (1.4)	Ref.	
Female	46 (11.5)	4 (8.7)	1.26 (0.42–3.79)		3 (6.5)	4.06 (0.98–16.82)		2 (4.4)	3.26 (0.61–17.28)	
**Period of diagnosis**			1.04 (0.92–1.18)			1.04 (0.83–1.29)			1.16 (0.90–1.51)	
2000–2003	147 (36.7)	6 (4.1)	Ref.		2 (1.4)	Ref.		0 (0.0)	–	
2004–2007	170 (42.4)	17 (10.0)	**2.61 (1.00–6.81)**		4 (2.4)	1.75 (0.32–9.68)		4 (2.4)	Ref.	

2008–2010	84 (20.9)	6 (7.1)	1.81 (0.56–5.79)		3 (3.6)	2.69 (0.44–16.40)		3 (3.6)	1.52 (0.33–6.99)
**Tobacco behaviour**									
Non-smoker	11 (2.7)	2 (18.2)	3.42 (0.70–16.82)	3.42 (0.70–16.82)	2 (18.2)	**13.15 (2.33–74.29)**	**20.11 (2.77–145.58)**	1 (10.0)	7.91 (0.83–74.81)
Former smoker	28 (7.0)	5 (17.9)	**3.35 (1.16–9.66)**	**3.35 (1.16–9.66)**	1 (3.6)	2.19 (0.25–18.87)	5.11 (0.51–50.86)	1 (3.6)	2.64 (0.30–23.38)
Smoker	361 (90.0)	22 (6.1)	Ref.	Ref.	6 (1.7)	Ref.	Ref.	5 (1.4)	Ref.
Unknown	1 (0.2)	0 (0.0)			0 (0.0)			0 (0.0)	
**Alcohol behaviour**									
Non-drinker	115 (28.7)	6 (5.2)	0.70 (0.27–1.79)		3 (2.6)	1.44 (0.34–6.13)		2 (1.7)	1.19 (0.21–6.59)
Former drinker	3 (0.7)	1 (33.3)	–		0 (0.0)	–		1 (33.3)	**33.63 (2.51–450.70)**
Drinker	274 (68.3)	20 (7.3)	Ref.		5 (1.8)	Ref.		4 (1.5)	Ref.
Unknown	9 (2.2)	2 (22.2)			1 (11.1)			0 (0.0)	
**Subsite**									
Proximal to oropharynx	97 (24.2)	7 (7.2)	1.00 (0.41–2.43)		4 (4.1)	2.57 (0.68–9.78)		3 (3.1)	2.42 (0.53–11.00)
Distal to oropharynx	304 (75.8)	22 (7.2)	Ref.		5 (1.6)	Ref.		4 (1.3)	Ref.
**Stage (7th edition TNM)**									
I	158 (39.4)	8 (5.1)	Ref.		3 (1.9)	Ref.		2 (1.3)	Ref.
II	98 (24.4)	8 (8.2)	1.67 (0.60–4.60)		2 (2.0)	1.08 (0.18–6.56)		1 (1.0)	0.80 (0.07–8.99)

III	77 (19.2)	8 (10.4)	2.17 (0.78–6.03)	2 (2.6)	1.38 (0.23–8.42)	3 (3.9)	3.16 (0.52–19.33)
IVa	63 (15.7)	5 (7.9)	1.62 (0.51–5.14)	2 (3.2)	1.69 (0.28–10.39)	1 (1.6)	1.27 (0.11–14.36)
IVb	5 (1.2)	0 (0.0)	–	0 (0.0)	–	0 (0.0)	–
IVc	0 (0.0)	–	–	–		–	
**Treatment**							
Only surgery	267 (66.6)	18 (6.7)	Ref.	7 (2.6)	0.85 (0.35–23.48)	6 (2.2)	
Surgery + others	107 (26.7)	9 (8.4)	1.27 (0.55–2.92)	1 (0.9)	Ref.	0 (0.0)	
No surgery	6 (1.5)	0 (0.0)	–	0 (0.0)	–	0 (0.0)	
Only radio	13 (3.2)	0 (0.0)	–	0 (0.0)	–	0 (0.0)	
Unknown	8 (2.0)	2 (25.0)		1 (12.5)		1 (12.5)	
**Histological diagnosis**							
SCC Conventional keratinizing	195 (48.6)	12 (6.2)	Ref.	5 (2.6)	Ref.	3 (1.5)	Ref.
SCC Conventional non keratinizing	151 (37.7)	13 (8.6)	1.44 (0.64–3.25)	2 (1.3)	0.51 (0.10–2.67)	2 (1.3)	0.86 (0.14–5.24)
SCC Other	50 (12.5)	3 (6.0)	0.97 (0.26–3.59)	1 (2.0)	0.78 (0.09–6.79)	1 (2.0)	1.31 (0.13–12.83)
Other non-SCC	5 (1.2)	1 (20.0)	3.81 (0.39–36.82)	1 (20.0)	9.5 (0.89–101.06)	1 (20.0)	**16.00 (1.35–189.21)**
**Tumour differentiation**							
Grade 1	106 (26.4)	3 (2.8)	Ref.	1 (0.9)	Ref.	0 (0.0)	

Grade 2	162 (40.4)	12 (7.4)	2.75 (0.76–9.98)	4 (2.5)	2.66 (0.29–24.11)	2 (1.2)	Ref.
Grade 3	128 (31.9)	14 (10.9)	**4.22 (1.18–15.09)**	4 (3.1)	3.39 (0.37–30.77)	5 (3.9)	3.23 (0.62–16.94)
Unknown	5 (1.2)	0 (0.0)		0 (0.0)		0 (0.0)	
**Total**	401 (100.0)	29 (7.2)		9 (2.2)		7 (1.8)	

LC: laryngeal cancer; OR: odds ratio; Distal to oropharynx: C02 and C02.0 and C02.1 and C02.2 and C02.3 and C03.1 and C04.1 and C04.9 and C06.0; Proximal to oropharynx: C02.8 and C02.9 and C05.8 and C06.2; [a] column percentage; [b] row percentage; [c] adjusted logistic regressions model using Backward Method initially including variables with crude p-value ≤ 0.25, and finally selecting variables with p-value < 0.05; bold represents statistically significant categories.

© 2020 by the authors. Licensee MDPI, Basel, Switzerland. This article is an open access article distributed under the terms and conditions of the Creative Commons Attribution (CC BY) license (http://creativecommons.org/licenses/by/4.0/).
